# Supplementary material for: R-loops acted on by RNase H1 influence DNA replication timing and genome stability in Leishmania
Source: Nat Commun. 2025 Feb 8;16:1470. doi: 10.1038/s41467-025-56785-y (PMC11807225; doi:10.1038/s41467-025-56785-y)
Supplement: Supplementary file 1 — Supplementary Information [file 41467_2025_56785_MOESM1_ESM.pdf]

SUPPLEMENTARY INFORMATION

R-loops acted on by RNase H1 influence DNA replication timing and genome stability in Leishmania

Jeziel D. Damasceno[1]*, Emma M. Briggs[2,3], Marija Krasilnikova[1], Catarina A. Marques[1], Craig Lapsley[1] and Richard McCulloch[1]*

1. The University of Glasgow Centre for Parasitology, The Wellcome Centre for Integrative Parasitology, University of Glasgow, School of Infection and Immunity, Sir Graeme Davies Building, 120 University Place, Glasgow, G12 8TA, United Kingdom.

2. University of Edinburgh, Institute for Immunology and Infection Research, School of Biological Sciences, Edinburgh, United Kingdom.

3. Biosciences Institute, Cookson Building, Newcastle University, Framlington Place, Newcastle upon Tyne, NE2 4HH, United Kingdom.

* To whom correspondence should be addressed:

jeziel.damasceno@glasgow.ac.uk

richard.mcculloch@glasgow.ac.uk

Supplementary Figure 1

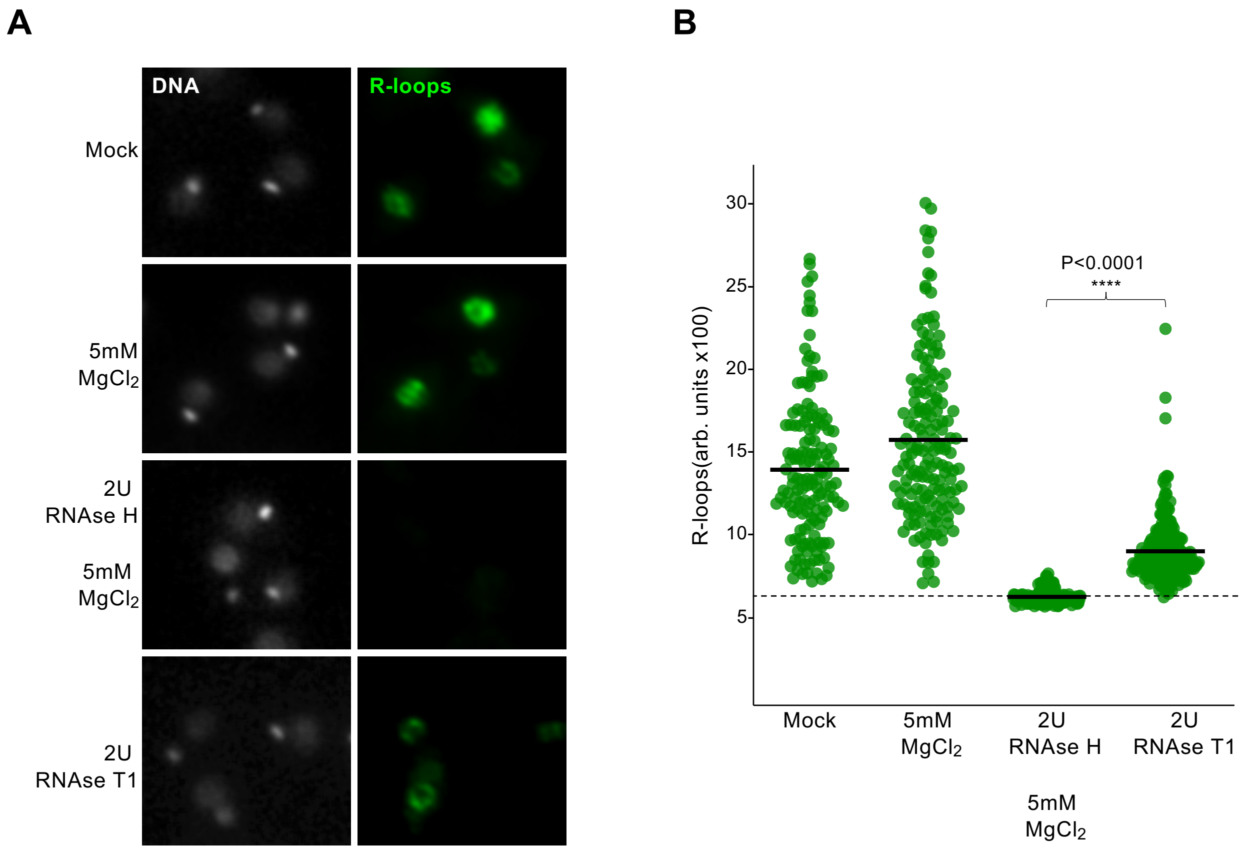

Supplementary Figure 1. Detection of R-loops in *Leishmania major* promastigote cells. A) Immunofluorescence analysis to detect R-loops in *L. major* wild type cells using S9.6 antibody; after fixing and permeabilization (see materials and methods), cells were resuspended in staining buffer and split into four aliquots; one aliquot remained with staining buffer only (mock) and the others received the indicated reagents; after incubation at 37 ºC for 1 h, cells were processed for immunofluorescence. **B)** Quantification of R-loops levels from A upon the indicated conditions, represented as arbitrary units (arb. units); black dotted line indicate background signal level based on average signal from RNAse H treated samples; statistic significance was determined by Kruskal-Wallis test (one-way ANOVA).

Supplementary Figure 2

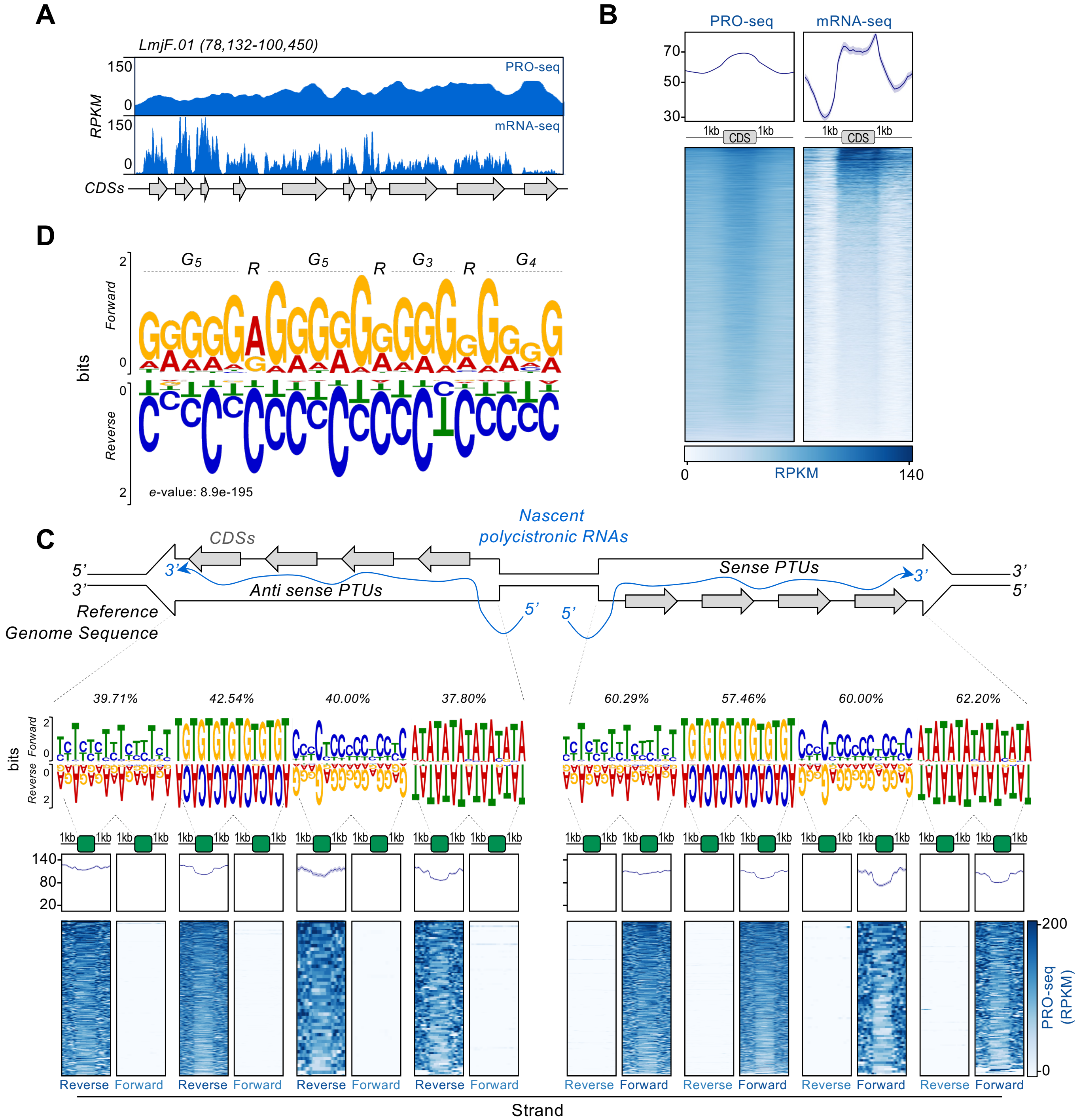

Supplementary Figure 2. Nascent and mature mRNA levels in *L. major*. A) Snapshot showing nascent (PRO-seq) and messenger (mRNA-seq) RNA in a representative genomic region; grey arrows at the bottom indicate annotated coding sequences (CDSs). **B)** Metaplots (top) and colormaps (bottom) showing global PRO-seq and mRNA-seq levels around CDSs; data was generated by *Grunebast et al* (*BioRXiv 10.1101/2023.11.23.568479*). **C)** R-loops peaks were grouped according to their sequence motif (logos on top of each colourmap), as identified by MEME in Figure 1D in the main text; R-loops peaks groups were further sub grouped according to the orientation (sense or antisense) of the polycistronic transcription units (PTUs) where they locate to; percentages show the proportion of indicated peak category located to a particular PTU type; metaplots and colourmaps show nascent transcripts (PRO-seq data generated by *Grunebast et al: BioRXiv 10.1101/2023.11.23.568479*) levels mapping the forward and reverse DNA strand. **D)** G-rich DNA sequence motif found in R-loop peaks, as identified by MEME analysis; the identified motif sequence $G_5RG_5RG_3RG_4$ resembles the consensus for G4s formation prone sequence $G_{\geq3}N_{1\text{-}7}G_{\geq3}N_{1\text{-}7}G_{\geq3}N_{1\text{-}7}G_{\geq3}N_{1\text{-}7}$; forward and reverse indicate motifs sequence as given by top and bottom strand, respectively, of reference genome.

Supplementary Figure 3

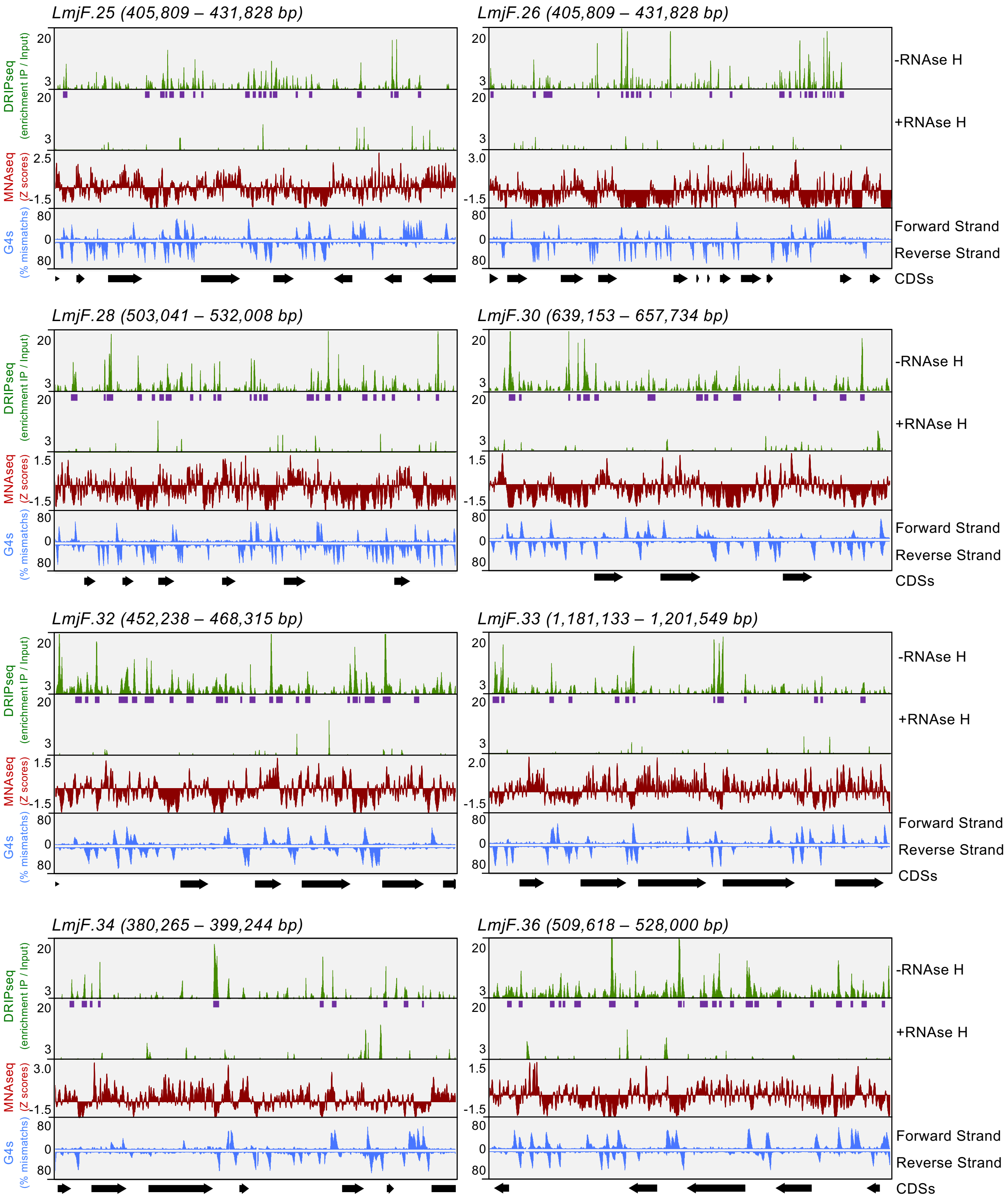

Supplementary Figure 3. Detection of R-loops around RNA Pol II transcribed regions in *L. major*. Snapshot of DRIP-seq at the indicated genomic regions; from top to bottom: track 1 and 2 (green), R-loop enriched regions relative to input material; -RNase H and +RNase H indicate mock or treatment with recombinant RNase HI prior to immunoprecipitation, respectively; R-loop peaks are indicated as purple bars bellow track 1; track 3 (dark red), MNase-seq data; track 4 (blue), mapping of G quadruplex structures (G4s); black arrows at the bottom of each panel indicate annotated coding sequences (CDSs).

Supplementary Figure 4

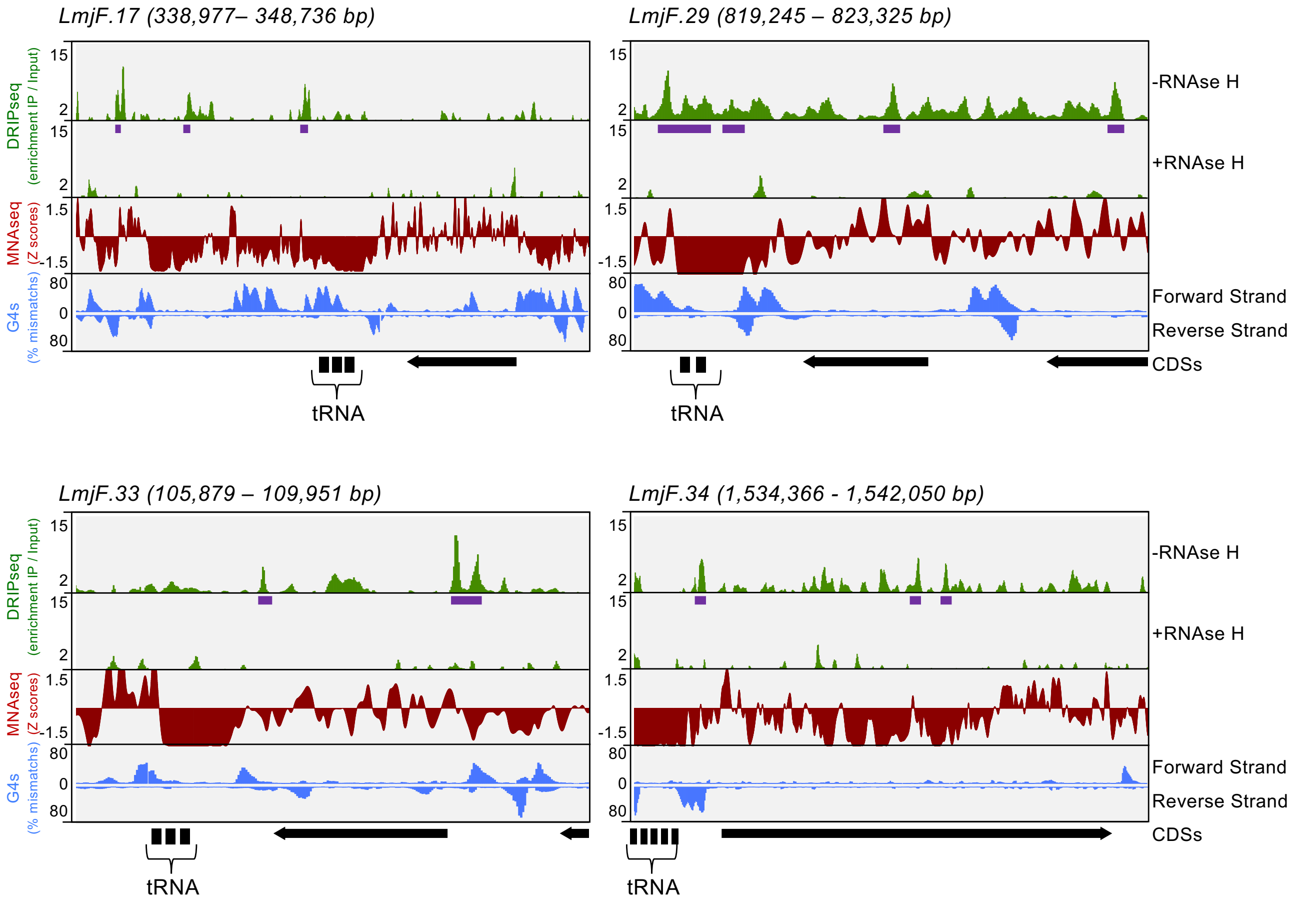

Supplementary Figure 4. Detection of R-loops around RNA Pol III transcribed regions in *L. major*. Snapshot of DRIP-seq at Pol III transcribed regions; from top to bottom: track 1 and 2 (green), R-loop enriched regions relative to input material; -RNase H and +RNase H indicate mock or treatment with recombinant RNase HI prior to immunoprecipitation, respectively; R-loop peaks are indicated as purple bars bellow track 1; track 3 (dark red), MNase-seq data; track 4 (blue), mapping of G quadruplex structures (G4s); black arrows at the bottom of each panel indicate annotated coding sequences (CDSs).

Supplementary Figure 5

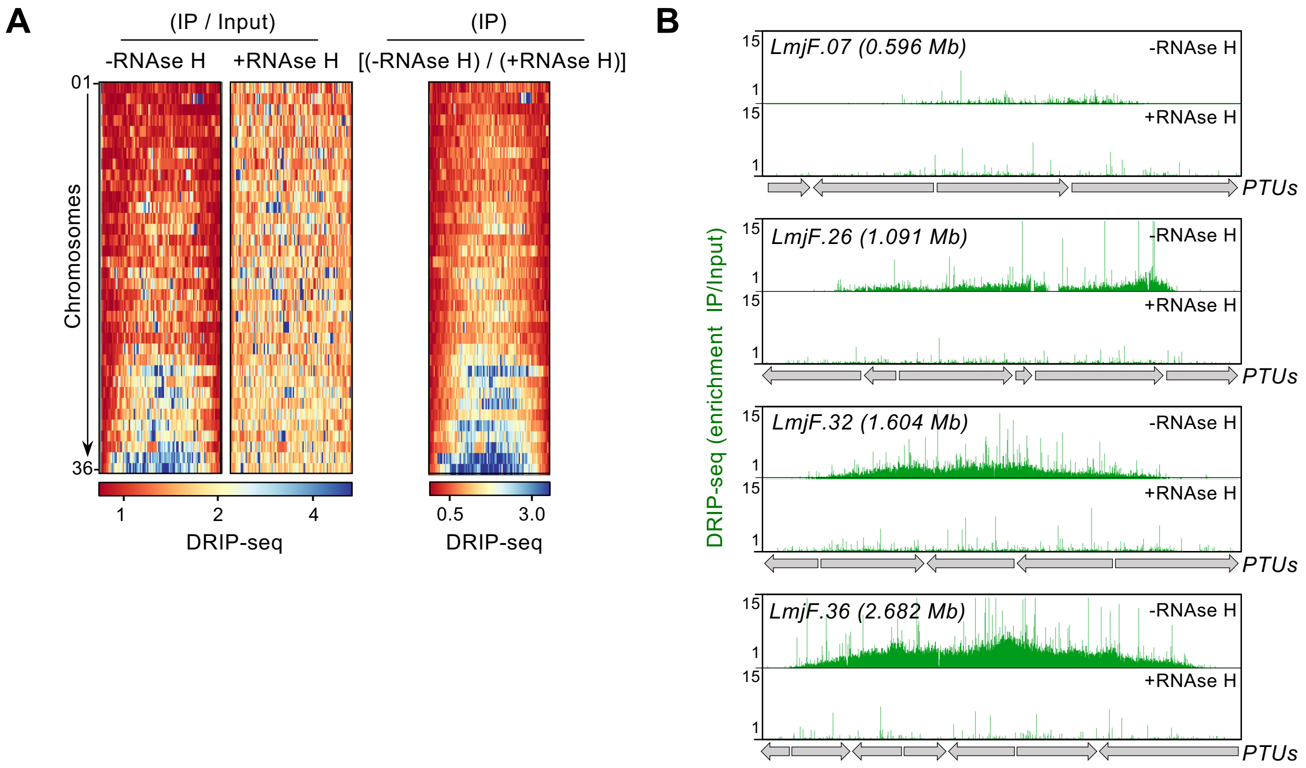

Supplementary Figure 5. Genome-wide R-loop distribution. A) Colourmap showing DRIP-seq in all 36 chromosomes of wild type (*WT*) cells; chromosomes are ordered by size; enrichment was calculated either as the enrichment of immunoprecipitated material relative to input (IP/Input; two panels at the left) or enrichment of immunoprecipitated material from mock relative to immunoprecipitated material from pre-treatment with recombinant RNase HI (IP; panel at right); -RNase H and +RNase H indicate mock or treatment with recombinant RNase HI prior to immunoprecipitation, respectively. **B)** Snapshot showing DRIP-seq in the indicated chromosomes; chromosome length is indicated in parenthesis; position and orientation of polycistronic transcription units (PTUs) is indicated for each chromosome.

Supplementary Figure 6

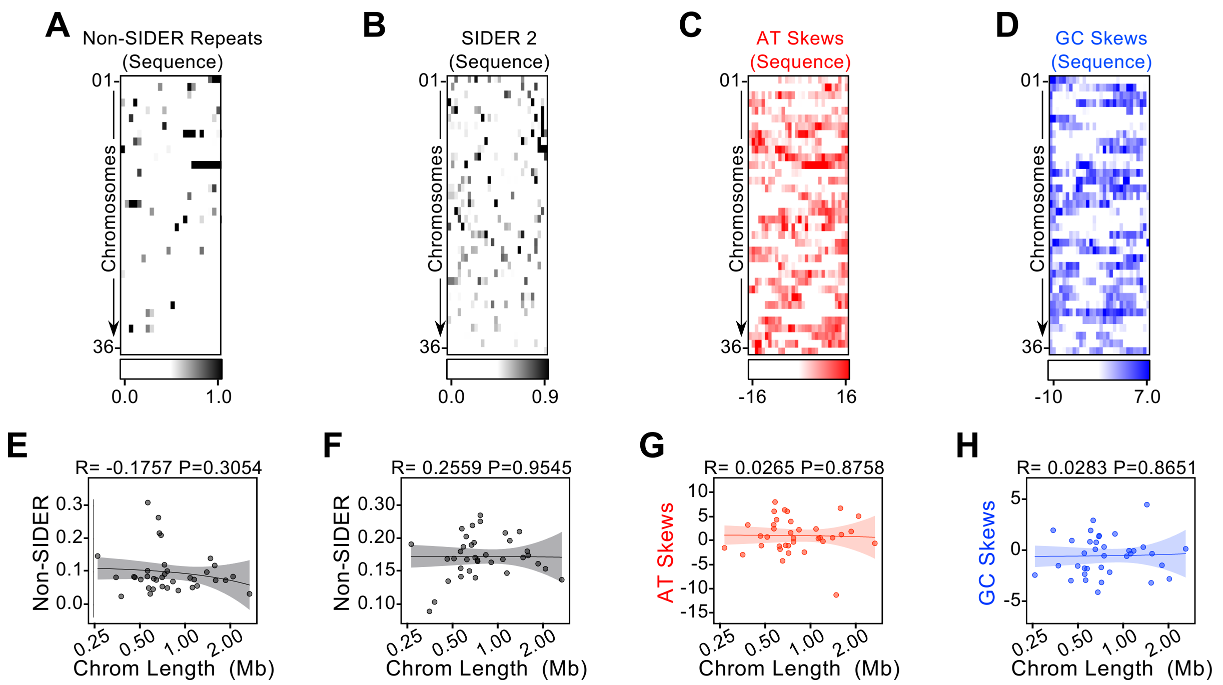

Supplementary Figure 6. Sequence content correlation with chromosome length. A), B), C) and **D)** Colourmaps showing density of the indicated genome features; chromosomes are ordered by size from top to bottom. **E), F), G)** and **H)** Simple linear regression analysis between chromosomes size and the indicated genomic features; R and *P* values are indicated at the top of each panel; shaded areas indicate 95% CI; lines indicate the best fit.

Supplementary Figure 7

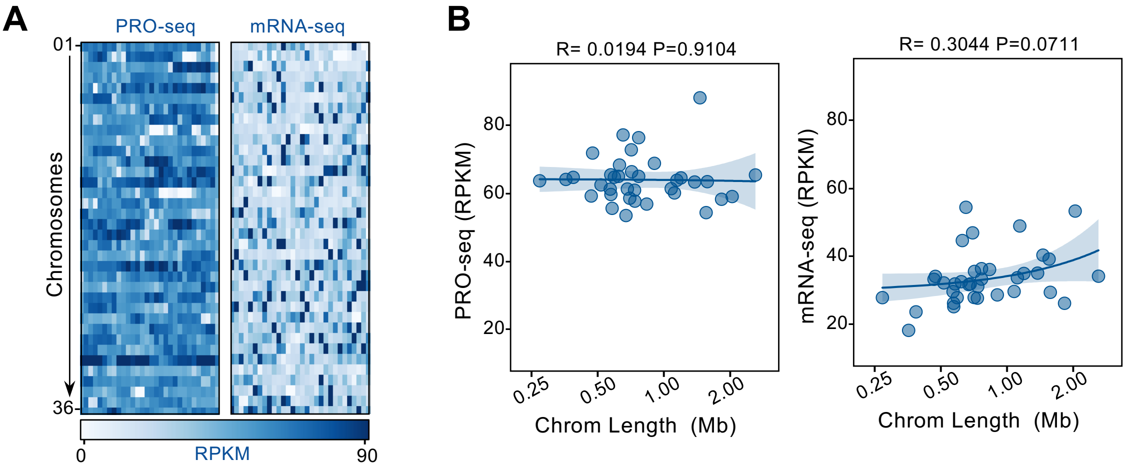

Supplementary Figure 7. Correlating RNA levels with chromosome length. A) Colourmaps showing nascent (PRO-seq) and messenger (mRNA-seq) levels in all chromosomes; chromosomes are ordered by size from top to bottom. **B)** Linear regression analysis between chromosomes size and PRO-seq or mRNA-seq average levels; R and *P* values are indicated at the top of each panel; shaded areas indicate 95% confidence interval; lines indicate the best fit.

Supplementary Figure 8

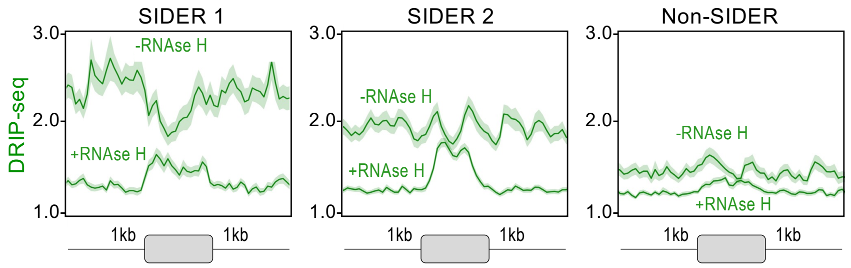

Supplementary Figure 8. Correlating R-loops with repeats in the *L. major* genome. Metaplots showing DRIP-seq signal around the indicated groups of repeats; lines indicate the mean and shaded areas represent the SEM.

Supplementary Figure 9

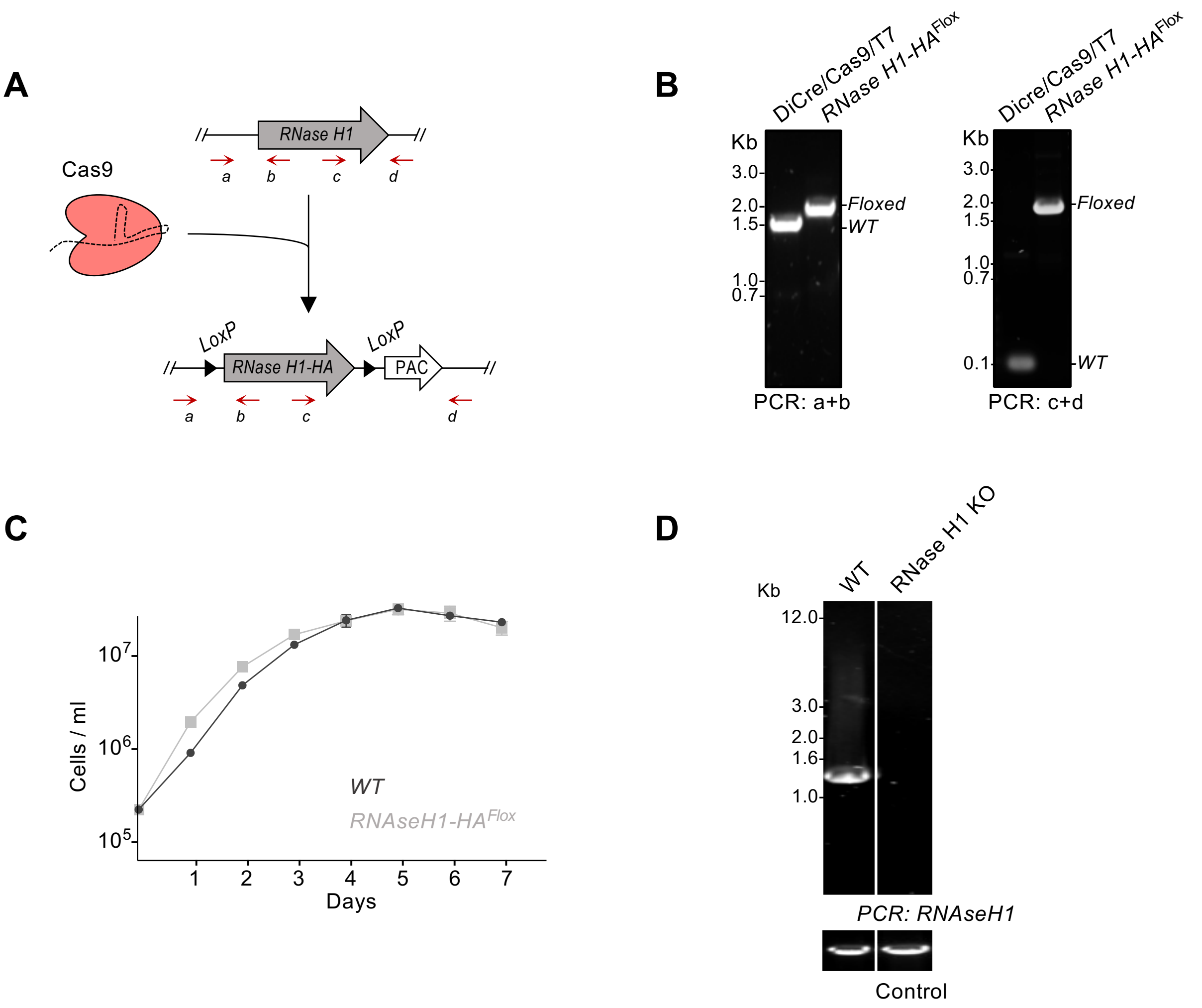

Supplementary Figure 9. Using CRISPR/Cas9 to generate an *RNaseH1-HA^{Flox}* cell line. A) CRISPR-Cas9 was used to flank the *RNase H1* ORF with *LoxP* sites and fuse it with HA tag (*RNase H1-HA^{Flox}*); *a, b, c* and *d* indicate the annealing position of primers used in B. **B)** PCR analysis of genomic DNA extracted from the DiCre/Cas9/T7-expressing cell line and the *RNase H1-HA^{Flox}* cell line; primer annealing positions are shown in A. **C)** Growth curve of *RNase H1-HA^{Flox}* cell line (grey) as compared to wildtype (*WT*) cells (black); cells were seeded at $2x10^5$ cells.mL^{-1} at day 0; cell density was assessed every 24 h, and error bars depict SEM. **D)** PCR analysis of genomic DNA extracted from *WT* cells and an *RNase H1 1 KO* clonal cell line; amplification of the *Rad51-3* gene was used as loading control.

Supplementary Figure 10

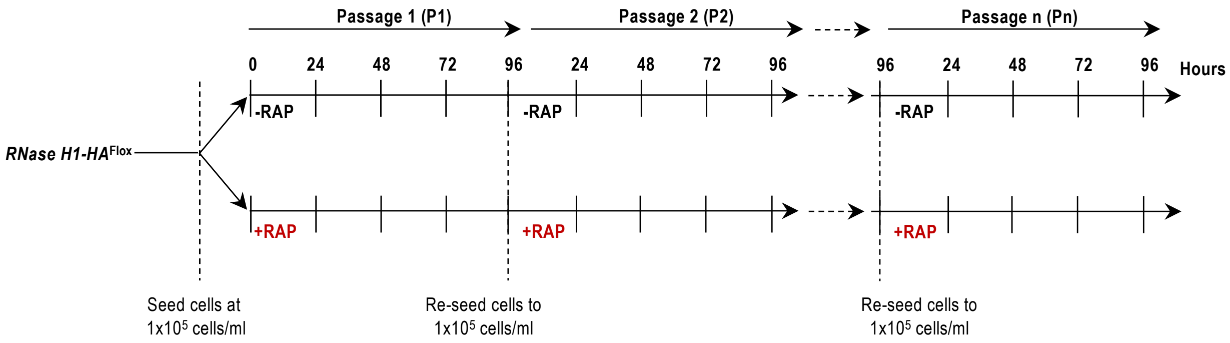

Supplementary Figure 10. **Workflow for KO induction in the *RNase H1-HA^{Flox}* cell cline**. Exponentially growing cells were seeded in medium with (+RAP) or without (-RAP) rapamycin; every 4 to 5 days of cultivation cells were re-seeded; all the experiments reported here were performed in cells subjected to this induction protocol.

Supplementary Figure 11

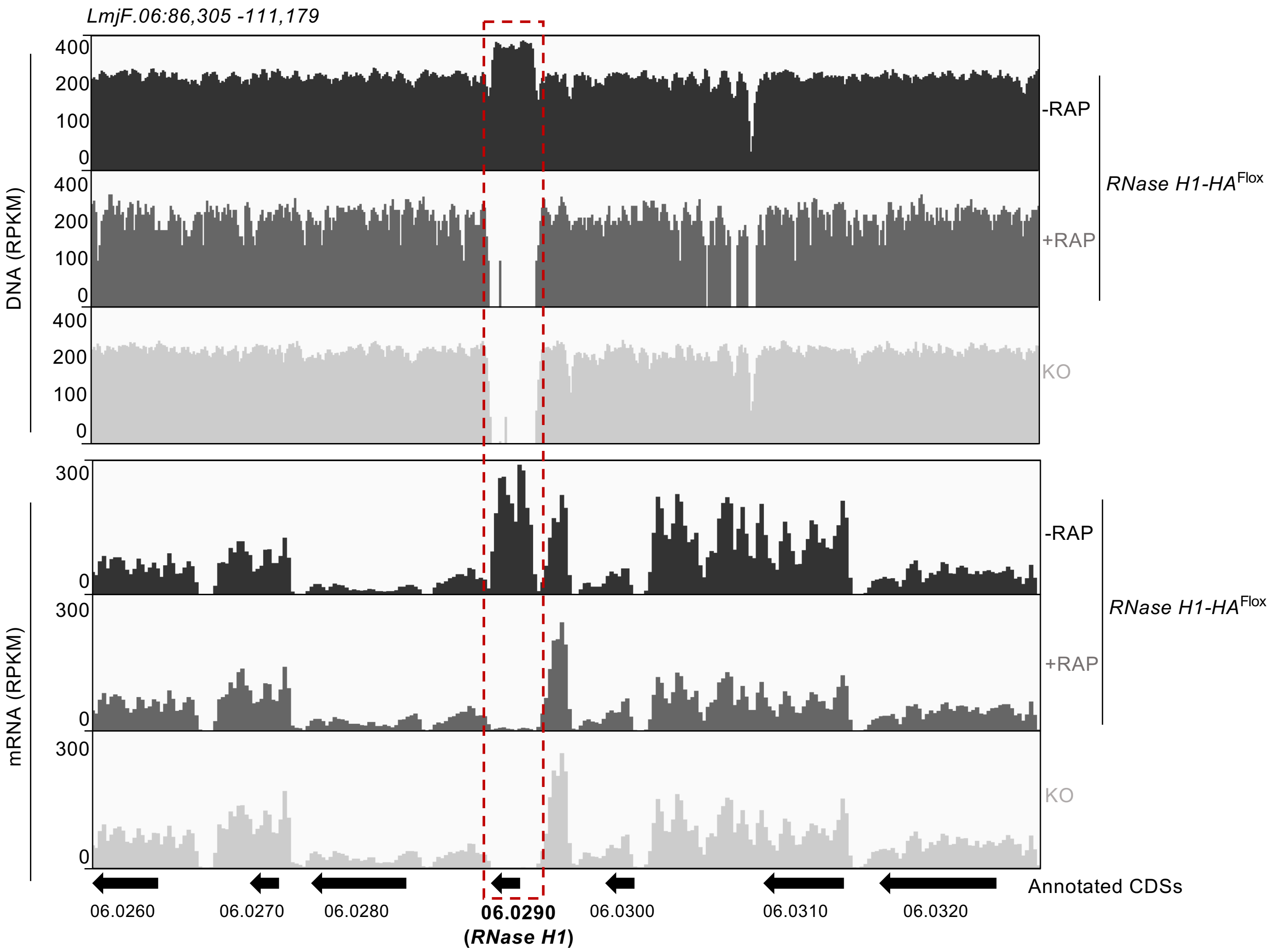

Supplementary Figure 11. Demonstration of *RNase H1* KO induction by whole genome sequencing. Whole genome DNA sequencing (top 3 panels) and whole genome RNA sequencing (bottom 3 panels)**;** dashed red line indicate position of RNase H1 gene position.

Supplementary Figure 12

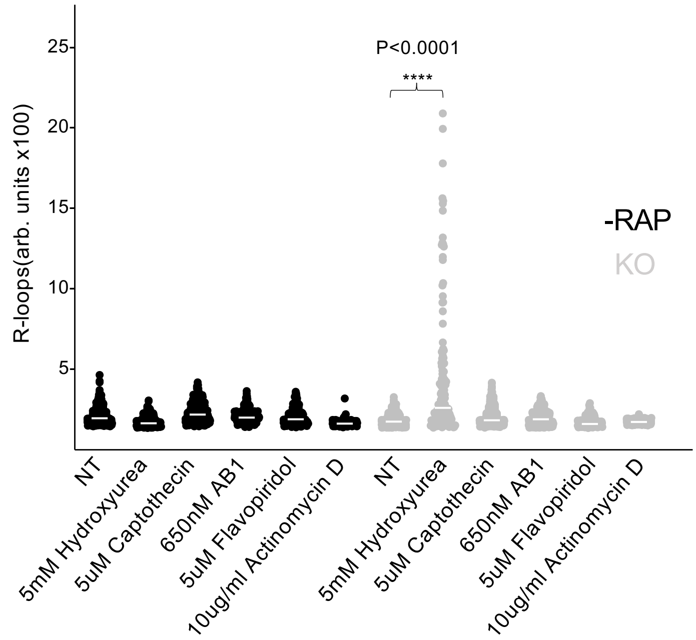

Supplementary Figure 12. Quantification of R-loop levels as detected via immunofluorescence using S6.9 antibody upon the indicated conditions; signal is represented as arbitrary units (arb. units); each treatment was performed for 6 hours; statistic significance was determined by Kruskal-Wallis test (one-way ANOVA).

Supplementary Figure 13

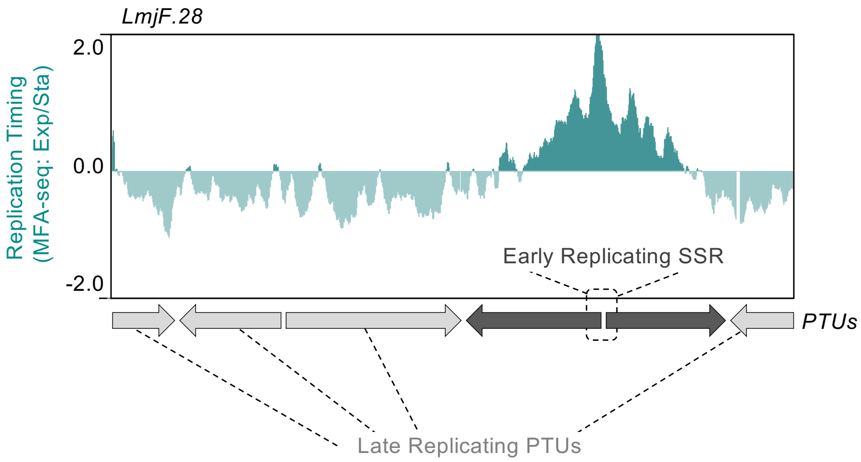

Supplementary Figure 13. **Determining replication timing from MFA-seq.** Replication timing profile (MFA-seq) for a representative chromosome of wild type cells; position and orientation of polycistronic transcription units (*PTUs*) is indicated at the bottom; SSRs overlapping the central region of an MFA-seq peak (positive values) were classified as early replicating SSRs; *PTUs* not surrounded by early replicating SSRs and overlapping with MFA-seq valleys (negative values), were classified as late replication PTUs.

Supplementary Figure 14

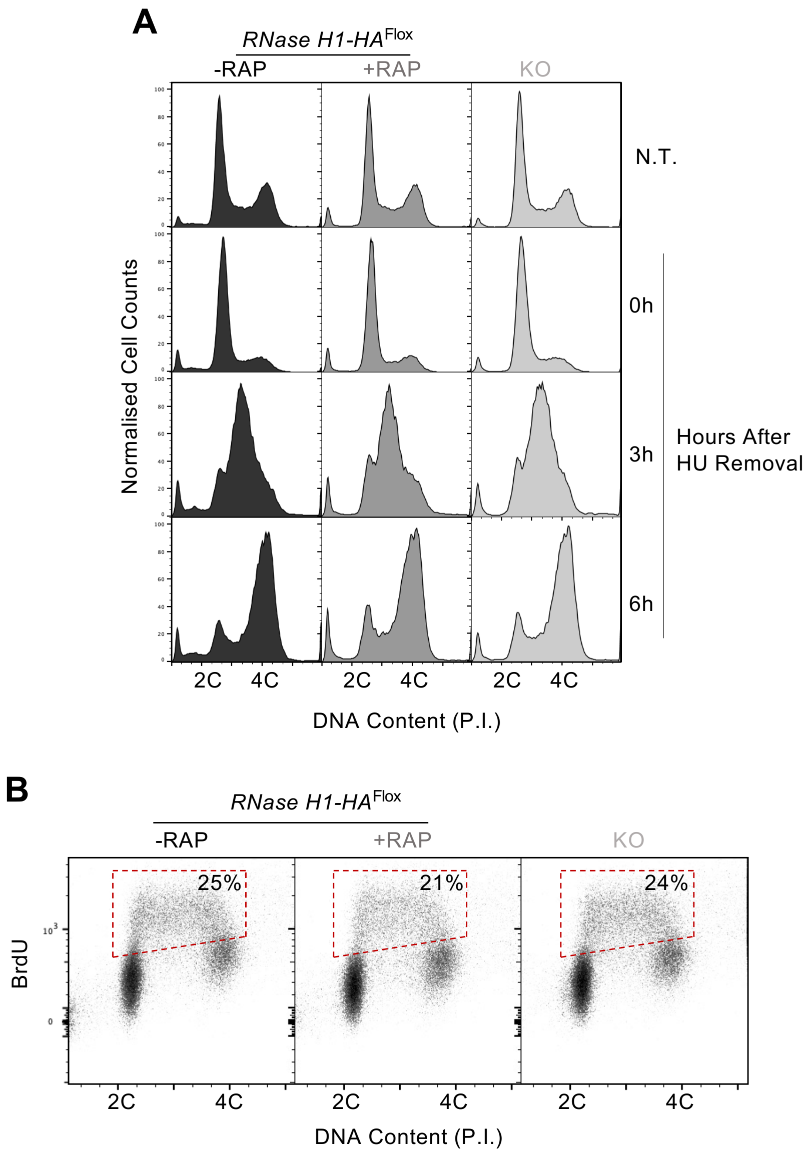

Supplementary Figure 14. Cell cycle progression analysis and quantification of S phase cells upon *RNase H1* KO. A) Exponentially growing cells were left untreated (N.T.) or treated for 8 hours with 5 mM hydroxyurea (HU) and then re-seeded in HU-free medium; cells were collected at the indicated time points after HU removal, fixed, stained with Propidium Iodide (P.I.) and analysed by FACS; 2C and 4C indicate one DNA content (G1) and double DNA content (G2/M), respectively. **B)** Exponentially growing cells were pulsed with BrdU for 30 minutes; BrdU fluorescence was detected under denaturing conditions using anti-BrdU antibody; 30,000 cells were analysed per condition; dashed red lines indicate the BrdU-positive population, i.e. cells in S phase; inset numbers indicate percentage of cells in S phase.

Supplementary Figure 15

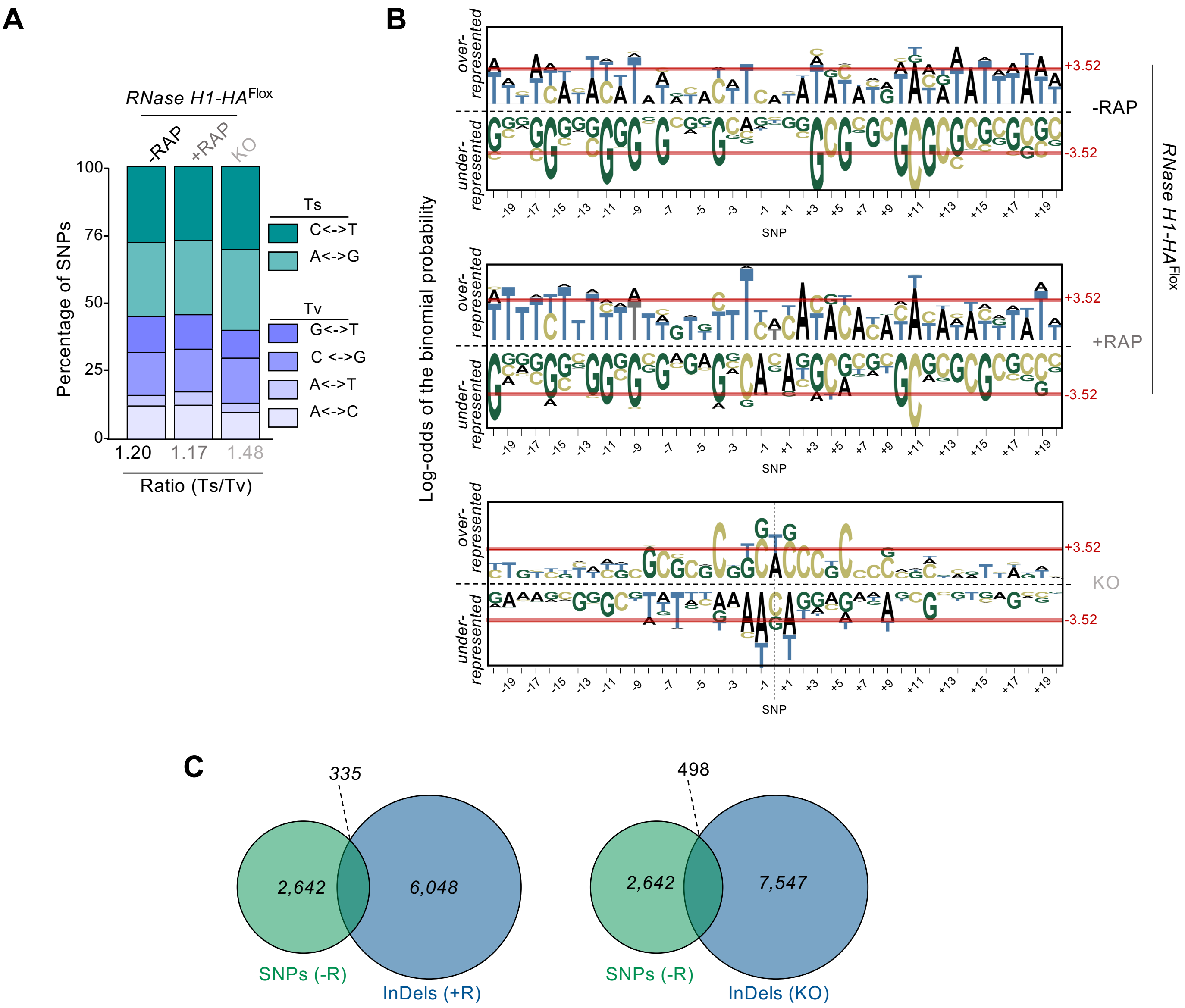

Supplementary Figure 15. Mutation signature analysis. A) Representation of the proportion of transition (Ts) and transversion (Tv) SNPs at the indicated conditions. **B)** SNP plus 20 flanking nucleotides was used for pLogo fold enrichment analysis; SNPs were centred in each plot (vertical dotted black line); overrepresented nucleotides are above, and underrepresented are below the horizontal dotted black line; horizontal red lines indicate threshold of significant enrichment ($p < 0.05$); font size indicates the enrichment magnitude. **C)** Venn diagrams showing the proportion of InDels events detected in induced (+R) and KO cells overlapping SNPs sites in uninduced cells (-R).
