# Supplementary material for: R-loops acted on by RNase H1 influence DNA replication timing and genome stability in Leishmania
Source: Nat Commun. 2025 Feb 8;16:1470. doi: 10.1038/s41467-025-56785-y (PMC11807225; doi:10.1038/s41467-025-56785-y)
Supplement: Supplementary file 2 — Reporting Summary [file 41467_2025_56785_MOESM2_ESM.pdf]

nature portfolio

Corresponding author(s): Richard McCulloch

Last updated by author(s): Jan 23, 2025

Reporting Summary

Nature Portfolio wishes to improve the reproducibility of the work that we publish. This form provides structure for consistency and transparency in reporting. For further information on Nature Portfolio policies, see our Editorial Policies and the Editorial Policy Checklist.

Statistics

For all statistical analyses, confirm that the following items are present in the figure legend, table legend, main text, or Methods section.

n/a	Confirmed	
☐	☒	The exact sample size (*n*) for each experimental group/condition, given as a discrete number and unit of measurement
☐	☒	A statement on whether measurements were taken from distinct samples or whether the same sample was measured repeatedly
☐	☒	The statistical test(s) used AND whether they are one- or two-sided<br>*Only common tests should be described solely by name; describe more complex techniques in the Methods section.*
☒	☐	A description of all covariates tested
☐	☒	A description of any assumptions or corrections, such as tests of normality and adjustment for multiple comparisons
☒	☐	A full description of the statistical parameters including central tendency (e.g. means) or other basic estimates (e.g. regression coefficient) AND variation (e.g. standard deviation) or associated estimates of uncertainty (e.g. confidence intervals)
☐	☒	For null hypothesis testing, the test statistic (e.g. *F*, *t*, *r*) with confidence intervals, effect sizes, degrees of freedom and *P* value noted<br>*Give P values as exact values whenever suitable.*
☒	☐	For Bayesian analysis, information on the choice of priors and Markov chain Monte Carlo settings
☒	☐	For hierarchical and complex designs, identification of the appropriate level for tests and full reporting of outcomes
☒	☐	Estimates of effect sizes (e.g. Cohen's *d*, Pearson's *r*), indicating how they were calculated

Our web collection on statistics for biologists contains articles on many of the points above.

Software and code

Policy information about availability of computer code

Data collection	Zeiss Elyra: https://www.zeiss.com/microscopy/en/products/light-microscopes/super-resolution-microscopes/elyra-7.html<br>Leica DMi8: https://www.leica-microsystems.com/products/light-microscopes/p/leica-dmi8/<br>DNBseq: https://www.bgi.com/global<br>Illumina: https://www.polyomics.gla.ac.uk/

Data analysis	GraphPad: https://www.graphpad.com/<br>Galaxy: https://usegalaxy.eu/<br>https://usegalaxy.org/<br>https://usegalaxy.fr/<br>https://usegalaxy.org.au/<br>BWA:https://github.com/lh3/bwa<br>DeepTools: https://deeptools.readthedocs.io/en/develop/<br>FastQC: https://github.com/s-andrews/FastQC<br>freeBayes: https://github.com/freebayes/freebayes<br>VCFtools: https://vcftools.sourceforge.net/<br>Rstudio: https://posit.co/download/rstudio-desktop/

For manuscripts utilizing custom algorithms or software that are central to the research but not yet described in published literature, software must be made available to editors and reviewers. We strongly encourage code deposition in a community repository (e.g. GitHub). See the Nature Portfolio guidelines for submitting code & software for further information.

Data

Policy information about availability of data

All manuscripts must include a data availability statement. This statement should provide the following information, where applicable:

- Accession codes, unique identifiers, or web links for publicly available datasets
- A description of any restrictions on data availability
- For clinical datasets or third party data, please ensure that the statement adheres to our policy

Sequences used in this study have been deposited in the EMBL-EBI European Nucleotide Archive, accession number PRJEB75366 (ERP159943); MFA-seq data is available in the NCBI Sequence Read Archive, accession number PRJNA1108605.

Research involving human participants, their data, or biological material

Policy information about studies with human participants or human data. See also policy information about sex, gender (identity/presentation), and sexual orientation and race, ethnicity and racism.

Reporting on sex and gender	n/a
Reporting on race, ethnicity, or other socially relevant groupings	n/a
Population characteristics	n/a
Recruitment	n/a
Ethics oversight	n/a

Note that full information on the approval of the study protocol must also be provided in the manuscript.

Field-specific reporting

Please select the one below that is the best fit for your research. If you are not sure, read the appropriate sections before making your selection.

[x] Life sciences [ ] Behavioural & social sciences [ ] Ecological, evolutionary & environmental sciences

For a reference copy of the document with all sections, see nature.com/documents/nr-reporting-summary-flat.pdf

Life sciences study design

All studies must disclose on these points even when the disclosure is negative.

Sample size	described in legends
Data exclusions	no exlcusions
Replication	all experiments were performed in at least two independent biological replicates; all were succesfsul
Randomization	described in figures
Blinding	n/a

Reporting for specific materials, systems and methods

We require information from authors about some types of materials, experimental systems and methods used in many studies. Here, indicate whether each material, system or method listed is relevant to your study. If you are not sure if a list item applies to your research, read the appropriate section before selecting a response.

Materials & experimental systems

n/a	Involved in the study
☐	☒ Antibodies
☐	☒ Eukaryotic cell lines
☒	☐ Palaeontology and archaeology
☒	☐ Animals and other organisms
☒	☐ Clinical data
☒	☐ Dual use research of concern
☒	☐ Plants

Methods

n/a	Involved in the study
☐	☒ ChIP-seq
☐	☒ Flow cytometry
☒	☐ MRI-based neuroimaging

Antibodies

Antibodies used	Mouse anti-HA (1: 5000, Sigma), mouse anti-EF1α (1: 40 000, Merck Millipore), anti-BrdU clone B44 (1: 500, BD Bioscience), and anti-DNA-RNA hybrid clone S9.6 (1:500, Sigma) primary antibodies were used here. Goat anti-Mouse IgG HRP-conjugated (ThermoFisher), goat anti-Mouse IgG Alexa Fluor 488-conjugated (ThermoFisher) and goat anti-Mouse IgG Alexa Fluor 594-conjugated (ThermoFisher) secondary antibodies were also used
Validation	All antibodies are commercial

Eukaryotic cell lines

Policy information about cell lines and Sex and Gender in Research

Cell line source(s)	All cell lines in this work were derived from Leishmania major strain LT252 (MHOM/IR/1983/IR)
Authentication	Cell lines were validated via PCR, whole genome sequencing and RNA-seq
Mycoplasma contamination	Cell lines did not test positive for Mycoplasm
Commonly misidentified lines (See ICLAC register)	Leishmania major strain LT252 (MHOM/IR/1983/IR) is not a commonly misidentified cell line

Plants

Seed stocks	n/a
Novel plant genotypes	n/a
Authentication	n/a

ChIP-seq

Data deposition

☒ Confirm that both raw and final processed data have been deposited in a public database such as GEO.

☒ Confirm that you have deposited or provided access to graph files (e.g. BED files) for the called peaks.

Data access links *May remain private before publication.*	Raw sequencing data files have been submitted to European Nucleotide Archive, accession number PRJEB75366 (ERP159943). Processed files have been submitted to GEO and accession numbers will be available soon.
Files in database submission	-bedgraph files for DRIP-seq and RNAse H1 ChIP-seq<br>-bed files with DRIP-seq peaks coordinates

Genome browser session (e.g. UCSC) | n/a

Methodology

Replicates	Two biological replicates
Sequencing depth	Approximately 30 million pair ended reads (100bp) per sample giving a read sequencing depth of approximately 180.
Antibodies	RNAse H1 ChIP-seq: rabbit anti-HA (Abcam, ab9110)<br>DRIP-seq: mouse anti-DNA-RNA hybrid clone S9.6 (Sigma, #MABE1095)
Peak calling parameters	No peaks calling performed for RNAse H1 ChIP-seq.<br>For DRIP-seq: enrichment of immunoprecipitated material over input is expressed as ratios and was determined using bamCompare (DeepTools) over a 60 bp rolling window. only reads with mapping quality >10 were considered. Peak calling was performed with in house script: peaks were identified based on regions exhibiting an enrichment greater than 2.5-fold in the DRIP material compared to the input control, as well as a more than 2.5-fold enrichment in samples untreated with RNase H compared to those treated with RNase H. Regions smaller than 60 bp were excluded from the analysis, and adjacent regions within 60 bp of each other were merged.
Data quality	Libraries sizes were normalized using the SES method, pair-ended extension was employed, PCR duplicates were ignored and regions were centred with respect to the fragment length.
Software	in house script

Flow Cytometry

Plots

Confirm that:

- [x] The axis labels state the marker and fluorochrome used (e.g. CD4-FITC).
- [x] The axis scales are clearly visible. Include numbers along axes only for bottom left plot of group (a 'group' is an analysis of identical markers).
- [x] All plots are contour plots with outliers or pseudocolor plots.
- [x] A numerical value for number of cells or percentage (with statistics) is provided.

Methodology

Sample preparation	Methanol or ethanol fixation
Instrument	BD FACSCelesta
Software	FlowJo: https://www.flowjo.com/
Cell population abundance	n/a
Gating strategy	n/a

- [ ] Tick this box to confirm that a figure exemplifying the gating strategy is provided in the Supplementary Information.
